# Genomic surveillance and evolution of co-circulating goose parvovirus and waterfowl circovirus in China

**DOI:** 10.1186/s13567-026-01737-7

**Published:** 2026-06-02

**Authors:** Xiaolong Lu, Meiqi Li, Qianqian Xu, Zhixin Xie, Yanhong Wang, Kaituo Liu, Wenhao Yang, Yu Chen, Ruyi Gao, Jiao Hu, Min Gu, Shunlin Hu, Xiaoquan Wang, Xiufan Liu, Xiaowen Liu

**Affiliations:** 1 Key Laboratory of Avian Bioproducts Development, Ministry of Agriculture and Rural Affairs, College of Veterinary Medicine, Yangzhou University, Yangzhou, China; 2 Jiangsu Co-Innovation Center for Prevention and Control of Important Animal Infectious Diseases and Zoonoses, Yangzhou University, Yangzhou, China; 3 Jiangsu Interdisciplinary Center for Zoonoses and Biosafety, Yangzhou University, Yangzhou, China; 4 Jiangsu Key Laboratory of Zoonosis, Yangzhou University, Yangzhou, China; 5 Joint International Research Laboratory of Agriculture and Agri-Product Safety of Ministry of Education of China, Yangzhou University, Yangzhou, China

**Keywords:** MGPV, NGPV, GoCV, DuCV, co-circulation, phylogenetic analysis

## Abstract

As the world’s largest waterfowl producer, China faces economic losses from Goose Parvovirus (GPV), worsened by waterfowl circovirus co-infections that increase pathogenicity and immunosuppression. However, current surveillance systems for these viruses lack systematization. This study conducted a comprehensive genetic analysis of GPV and waterfowl circovirus to identify genomic characteristics and recombination events. Surveillance analysis of GPV revealed a significant host-associated genotypic divergence from 2018 to 2024 in China. Goose isolates were predominantly the Mutated GPV (MGPV, 87%), while duck isolates were mainly the duck-adapted Novel GPV (NGPV, 88%). This divergence was corroborated by a global analysis, which confirmed high intra-clade similarity but substantial overall genetic diversity. Whole-genome recombination analysis revealed one isolate as a unique NGPV recombinant, with an NGPV strain as the major parent and an Early GPV strain as the minor parent. Furthermore, our study identified a persistent, host-specific co-circulating GPV and waterfowl circovirus: MGPV/GoCV in geese and NGPV/DuCV in ducks. Phylogenetic analysis shows that waterfowl circovirus exhibits significant genetic diversity: GoCV has two lineages (GoCV-I prevalent in China, GoCV-II with cross-species transmission to European Anser anser), while DuCV has three genotypes with distinct geographical distributions and host ranges across Asia and North America. Genomic analysis corroborated these phylogenetic findings and indicated ongoing genetic variation and recombination as key drivers of waterfowl circovirus evolution. In conclusion, this study systematically elucidates the ongoing adaptive evolution and genetic plasticity of GPV and waterfowl circovirus, providing a scientific basis for targeted waterfowl disease prevention strategies.

**Supplementary Information:**

The online version contains supplementary material available at 10.1186/s13567-026-01737-7.

## Introduction

China contributes more than half of the global waterfowl production, establishing itself as a paramount producer in the industry [1]. Goose Parvovirus (GPV) is an important waterfowl pathogen that can cause Derzsy’s disease, a fatal condition in goslings and ducklings, seriously endangering the development of waterfowl farming [2, 3]. It can cause symptoms such as loss of appetite, mental fatigue, intestinal bleeding, watery diarrhea, and even high mortality in goslings and ducklings [4]. Furthermore, GPV can spread both horizontally and vertically, thereby broadening infection sources and exacerbating the challenge of controlling Derzsy’s disease [5]. Therefore, GPV has long been a significant concern in the poultry industry, especially in waterfowl farming. GPV is a non-enveloped, single-stranded DNA virus with a genome of approximately 5.1 kb that can anneal to form double-stranded DNA [6]. The coding region of GPV genome, situated in the genome’s central region, contains two open reading frames (ORFs) encoding five proteins. The left ORF encodes non-structural proteins NS1 and NS2, while the right ORF encodes structural proteins VP1, VP2, and VP3 [7]. As the primary structural protein, VP1 is essential for capsid assembly and viral replication. Critically, specific mutations at residues 164 K, 165 K, and 167 K are indispensable for goose parvovirus proliferation, likely by modulating these core functions [8]. VP2 and VP3 are the primary immunogenic proteins, inducing neutralizing antibodies. VP2 can form virus-like particles for immunization, while VP3, the most abundant capsid protein, serves as an ideal antigen for GPV serological detection, crucial for diagnosing and preventing Derzsy’s disease [9–11].

Since its first discovery in China in 1956 and isolation from goose embryos in 1961, GPV has been reported in numerous countries worldwide, including the UK, Poland, Sweden, Hungary, and many others [12–15]. As of now, GPV exhibits substantial genetic diversity, evolving into several branches. These branches represent distinct GPV genotypes formed during evolution: Classical GPV typically refers to historical, highly pathogenic strains; Early GPV designates primordial isolates; Attenuated GPV comprises strains attenuated through laboratory adaptation; Mutated GPV (MGPV) involves variants with altered antigenicity or pathogenicity due to mutation; and Novel GPV (NGPV) denotes recently identified strains with unique genetic characteristics, often associated with Short Beak and Dwarfism Syndrome (SBDS). In addition, Muscovy duck parvovirus (MDPV), which primarily infects Muscovy ducks, is another distinct waterfowl pathogen [16, 17]. In China, Classical GPV and NGPV are the predominant genotypes circulating among waterfowl [16, 18]. NGPV is derived from Classical GPV, yet they exhibit vastly different pathogenicity. Classical GPV typically induces classic symptoms in waterfowl, such as diarrhea, intestinal bleeding, and systemic multi-organ lesions. In contrast, NGPV primarily affects ducklings, impairing skeletal development and causing SBDS [19]. GPV primarily infects goslings and various ducklings. However, recent years have witnessed occasional cross-species transmission, expanding the host range to include birds such as swans and ostriches [20–22]. Mixed infections, often involving viral interactions that enhance pathogenicity or suppress immunity, lead to more severe disease outcomes in waterfowl than single infections. Specifically, waterfowl circovirus induces significant immunosuppression mechanistically by targeting and damaging lymphoid organs, such as the bursa of Fabricius and thymus. This process leads to the depletion of lymphocytes and the impairment of critical immune responses, thereby creating a permissive environment for secondary pathogens [23]. A key example is the frequent co-infection of GPV with immunosuppressive waterfowl circovirus, a combination that poses a significant threat to the industry. For instance, in geese, co-infection with Goose Circovirus (GoCV) exacerbates diseases like gosling feather loss and goose broke feather disease [24]. Similarly, in ducks, a high co-infection rate between NGPV and Duck Circovirus (DuCV) amplifies viral replication, intensifies clinical symptoms, and increases overall pathogenicity [25, 26].

Given the severe impact of GPV, waterfowl circovirus, and their mixed infections on waterfowl farming, a comprehensive epidemiological investigation and analysis is urgently needed. In this study, we performed epidemiological investigations across China and conducted phylogenetic analyses on the isolated strains using a suite of bioinformatics tools. Furthermore, by analyzing publicly available virus sequences from global databases, we performed a multidimensional analysis to elucidate their worldwide prevalence patterns. Taken together, this study will provide valuable insights into the genetic evolution, distribution, and mixed infections of GPV and waterfowl circovirus, thereby informing more effective control measures.

## Materials and methods

### Sample information and DNA extraction

Tissue and organ samples were collected from diseased geese and ducks from waterfowl farms and free-range populations across China, including provinces Jiangsu, Anhui, Jiangxi, Shandong, and Guangdong. The tissues and organs were homogenized in a double-antibody PBS solution, centrifuged at 8000 rpm for 5 min at 4 °C, and the supernatant was subsequently extracted. DNA extraction was performed using the MagaBio Plus Virus DNA/RNA Purification Kit III (Bioer, Hangzhou, China) following the manufacturer’s instructions.

### Amplification and sequencing of GPV and waterfowl circovirus

Primers were designed using SnapGene software based on the NCBI GPV, GoCV, and DuCV sequence, with the primer information detailed in Additional file 1. DNA was extracted from diseased tissues and screened for GPV, GoCV, and DuCV by PCR. The reaction system is detailed in Additional file 2. The complete genomes of GPV, GoCV, and DuCV were amplified using a segmented PCR strategy with specific primer pairs. Briefly, the GPV genome was amplified in six overlapping fragments, while the GoCV and DuCV genomes were each amplified in two overlapping fragments. All PCRs were conducted using 2 × Phanta Flash Master Mix (Vazyme, Nanjing, China) under the following universal cycling conditions: initial denaturation at 98 °C for 1 min; 32 cycles of 98 °C for 10 s, primer-specific annealing, and 72 °C for a variable extension time (Additional file 3); and a final extension at 72 °C for 1 min. The Sanger sequencing products from each fragment were assembled into a consensus genome sequence for each virus using SeqMan software (DNASTAR, Inc., WI, USA). PCR products were separated on a 1% agarose gel, and target bands were excised, purified, and sent to Qingke Biotechnology (Beijing, China) for sequencing.

### Phylogenetic analysis

The complete gene and VP gene sequences of the reference viruses were retrieved from the NCBI public database. Details of the isolated strains in this study were summarized in Additional file 4. Multiple sequence alignment was conducted using MAFFT 7.0 software [27], followed by sequence cleaning to eliminate aberrant, excessively long or short, and low-similarity sequences, thereby enhancing data quality. ModelFinder implemented in IQ-TREE 2 software [28] was employed to validate phylogenetic analysis models, identifying the most appropriate model for describing sequence evolution. A phylogenetic tree was subsequently constructed using the Maximum Likelihood method in IQ-TREE 2 software to elucidate the evolutionary relationships among the sequences. The final phylogenetic tree was generated and visualized using FigTree v.1.4.4 software [29] and iTOL software [30].

For waterfowl circoviruses (Goose circovirus, GoCV, and Duck circovirus, DuCV; family *Circoviridae*): We applied the well-defined quantitative framework established for waterfowl circoviruses [31]. Distinct viral species were recognized when whole-genome nucleotide identity was below 80%, reflecting the substantial divergence between known species such as DuCV and GoCV. Genotypes were defined using a p-distance threshold of > 0.17 (corresponding to nucleotide identity < 83%) in whole-genome pairwise sequence comparison (PASC) analysis, following the convention in the field. Within each genotype, lineages were delineated as monophyletic clusters with strong statistical support (bootstrap value > 70%) and evidence of distinct spatiotemporal distribution. For Goose parvovirus (GPV), its status as a distinct viral species (a member of the genus *Parvovirus* within the family *Parvoviridae*) has been established based on its serological properties, host range, and pathogenicity. Therefore, in this study, we adhered to this established taxonomic framework [32]. Our focus was placed on analyzing the genetic diversity within this species. Within the GPV species, major genotypes were defined as phylogenetic clusters with genome nucleotide identity typically ranging from 95 to 99%. Distinct lineages were identified as well-supported sub-clusters (bootstrap support > 70%) within a genotype, characterized by higher sequence conservation (nucleotide identity > 96%).

### Nucleotide homology analysis

To evaluate the genetic relatedness of GPV or waterfowl circovirus, we conducted a systematic homology analysis of their nucleotide sequences with BioAider V1.532 software (Institute of Microbiology, CAS, China), respectively. This process involved multi-sequence alignment to precisely quantify the degree of sequence similarity. These data provide a foundation for inferring their evolutionary relationships and taxonomic classification. Final visualization was performed using Chiplot software [33].

### Recombination analysis

Recombination events were detected using seven methods from the Recombination Detection Program (RDP4.0) toolkit: RDP, CHIMAERA, 3SEQ, MAXCHI, GENECONV, BootScan, and SiScan [34]. Each method offers a unique perspective on potential recombination. A stringent criterion was applied, considering a recombination event valid only if detected by at least four methods, thereby minimizing false positives and enhancing test accuracy. Detected recombination events were further validated using SimPlot 3.5.1 software (MD, USA), which offers visual evidence to confirm the authenticity and precise location of these events.

## Results

### Genetic evolution and global epidemiology of GPV

**Figure 1:**
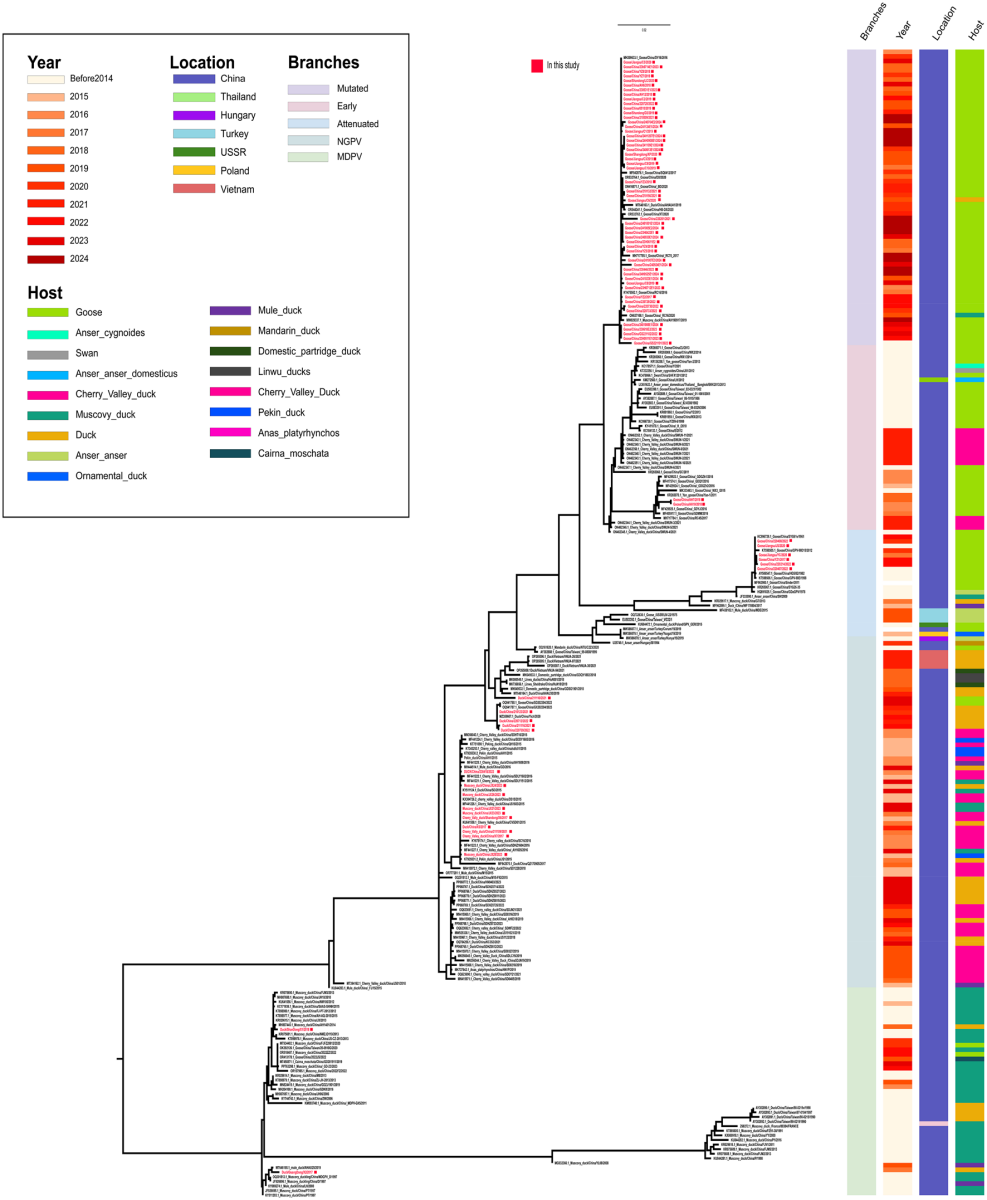
**Phylogenetic analysis of the GPV VP1 gene**. Phylogenetic tree was constructed by GPV VP1 gene. Red dots indicate the GPV isolates from this study. The color-coded bars to the right of the tree denote the corresponding branches, isolation time, geographic location, and host for each strain.

**Figure 2:**
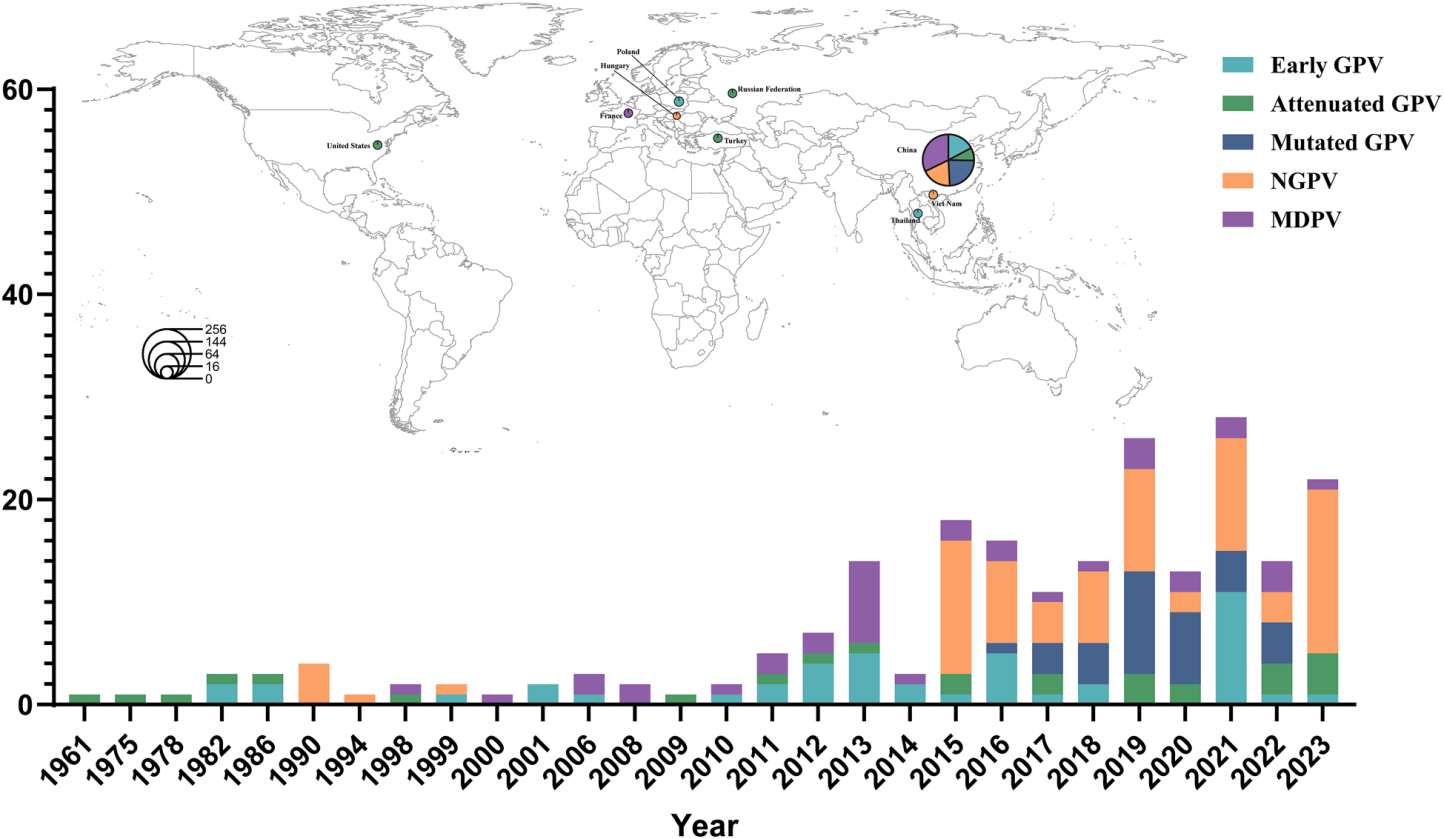
**Global distribution and genetic evolution of GPV**. Spatiotemporal distribution and genetic diversity of GPV and related strains isolated from 1961 to 2023. The depicted strains include Early GPV, Attenuated GPV, MGPV, NPGV, and MDPV in the bar chart. The Y-axis indicates the number of isolates, and the X-axis represents the year of isolation. The map illustrates the global distribution of countries reporting GPV outbreaks, including China, Thailand, Hungary, France, the United States, Poland, Turkey, Vietnam, and the Russian Federation. The accompanying pie chart details the proportional composition of GPV types: Early GPV (teal), Attenuated GPV (green), MGPV (navy blue), NGPV (orange), and MDPV (purple).

GPV poses a significant threat to China’s waterfowl industry. Our laboratory conducted epidemiological surveillance from 2018 to 2024, isolating 60 goose-derived and 17 duck-derived GPV strains. Phylogenetic analysis based on the VP1 gene–encoding the major capsid protein with both conserved and variable regions–revealed distinct patterns. Among goose isolates, 87% (52/60) belonged to the MGPV lineage, compared to 10% (6/60) Attenuated GPV and 3% (2/60) Early GPV. In contrast, duck isolates were predominantly NGPV (88%, 15/17), with only 2 MDPV strains identified (Figure 1). This suggests GPV is adapting to ducks, with distinct dominant genotypes in duck- versus goose-derived strains. We analyzed the global prevalence trend of GPV using sequences from the NCBI public database. The VP3 gene was selected as the target for our global prevalence analysis of GPV due to its dual significance: it is both a key gene in the virus’s genetic evolution and the sequence with the most comprehensive data available on NCBI. The results confirmed the rise of MGPV (48%, 61/127) since 2016, largely replacing Early GPV (1961–2016) in geese. The prevalence of NGPV/MDPV in duck pointed to host adaptation. In China, a major waterfowl producer, GPV showed regional differentiation. Briefly, MGPV was prevalent in key goose–rearing areas like Jiangsu, while NGPV dominated in duck-rearing regions such as Shandong and Guangdong. Moreover, GPV isolates displayed a multi-branched genetic structure in China, primarily comprising the Classical GPV and NGPV lineages. The Classical GPV lineage further split into Early GPV, Attenuated GPV, and MGPV, illustrating significant genetic diversity. In contrast, GPV outbreaks in Europe and Southeast Asia tend to be sporadic (Figure 2).

**Figure 3:**
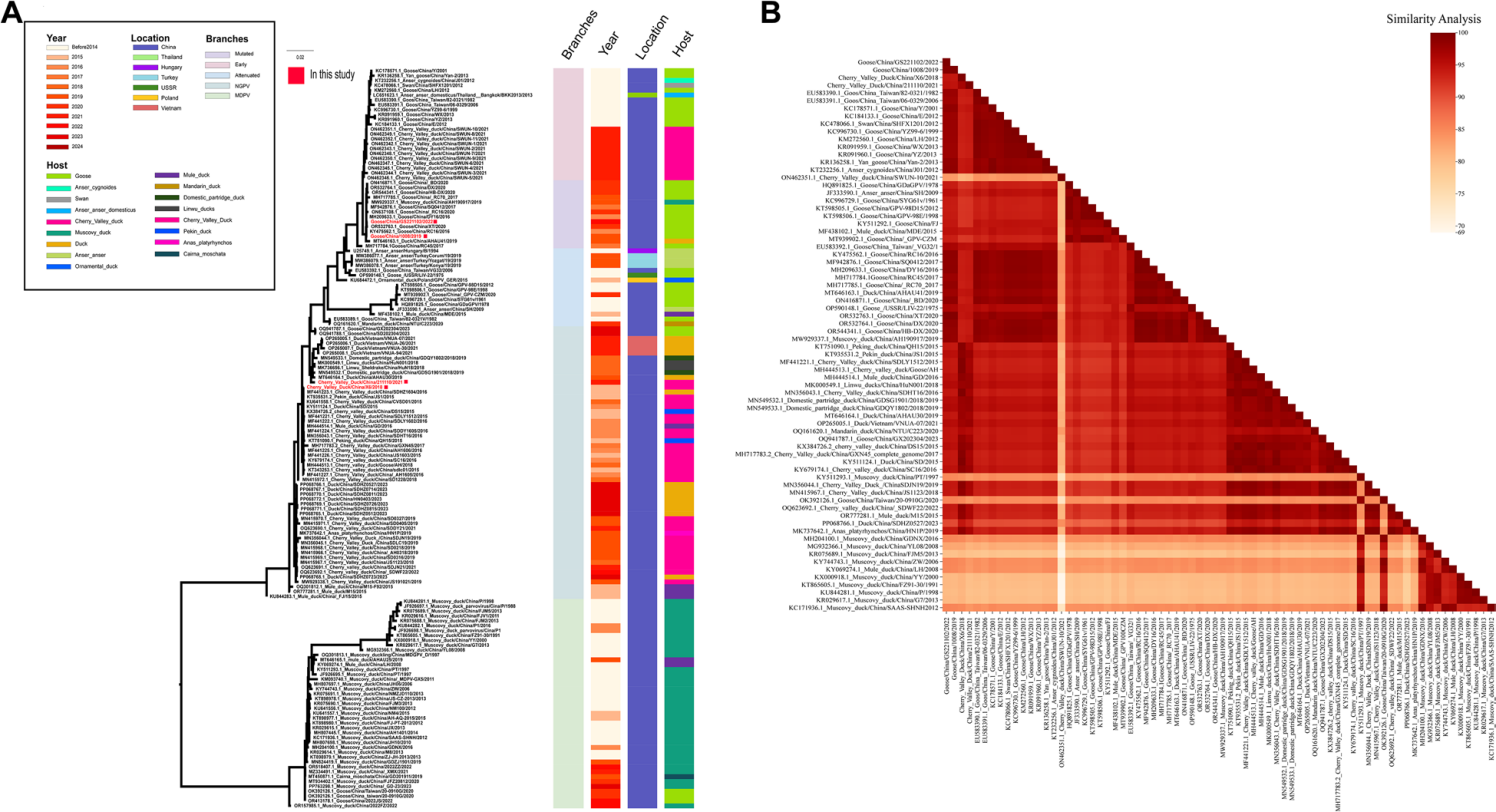
**Analysis of whole-genome evolution and genomic nucleotide similarity of GPV**.** A** Phylogenetic analysis of GPV based on whole-genome sequences was performed. Red dots indicate the four GPV isolates sequenced in this study. The color-coded bars to the right of the tree denote the corresponding branches, isolation time, geographic location, and host for each strain. **B** Genomic nucleotide similarity analysis of GPV. Nucleotide similarity analysis of the complete genomes from four representative GPV strains isolated in this study and reference strains. The heatmap displays pairwise nucleotide similarity percentages, with the color gradient scale (top right) indicating values ranging from 69 to 100%.

Additionally, four isolates obtained from our laboratory were subjected to whole-genome genetic evolution analysis. The results indicated that the evolutionary trends of the whole genome and the VP1 gene were largely consistent. Based on this whole-genome analysis, GPV primarily divides into two major branches: Classical GPV and NGPV. Classical GPV further comprises three sub-lineages: Early GPV, Attenuated GPV, and MGPV. MGPV has emerged as a prevalent variant in geese in recent years, exemplified by the strains Goose/China/GS221102/2022 and Goose/China/1008/2019 isolated in this study. NGPV, representing a novel branch derived from GPV, predominantly occurs in duck-derived isolates [e.g., Cherry Valley duck/211110/2021 and Cherry Valley duck/X6/2018 from our laboratory], suggesting GPV’s gradual adaptation to duck hosts. Our analysis also revealed that Early GPV was dominant in geese prior to 2016, whereas MGPV became the prevailing genotype from 2016 onwards. NGPV began spreading within the Cherry Valley duck population in 2015 and subsequently became the predominant genotype in ducks (Figure 3A).

### Genomic nucleotide similarity analysis of GPV

To analyze the genetic variation of GPV epidemic strains in China, we compared the nucleotide consistency of two representative MGPV strains (Goose/China/GS221102/2022 and Goose/China/1008/2019) and two representative NGPV strains (Cherry Valley duck/211110/2021 and Cherry Valley duck/X6/2018) with 73 GPV reference strains from NCBI. The results showed that Goose/China/GS221102/2022 and Goose/China/1008/2019 exhibited the highest nucleotide similarity (96.62%-99.59% and 96.4%-99.57%, respectively) with the MGPV branch (13 strains). Similarly, Cherry Valley duck/211110/2021 and Cherry Valley duck/X6/2018 showed the highest similarity (95.75%-98.28% and 96.25%-99.81%, respectively) with the NGPV branch (17 strains). Globally, the nucleotide similarity across GPV genomes ranged widely from 79.01% to 99.98%. To address potential genetic heterogeneity, we stratified the host sources in the Figure 3B into three primary categories: Goose (including derivatives), Duck (encompassing domestic, Cherry Valley, Pekin, and mule ducks), and Muscovy duck. Identical sequences (100% identity) were excluded to focus on genomic diversity. The analysis revealed distinct nucleotide identity ranges within each group (Goose: 83.48%–99.98%; Duck: 73.38%–99.92%; Muscovy duck: 78.99%–99.67%). More importantly, the inter-group comparisons showed substantial overlap and broad variability, particularly between Duck and Muscovy duck (ranging as low as 69.00%). Collectively, these findings demonstrate substantial genomic variability across different waterfowl species (Figure 3B).

### Whole-genome recombination analysis of GPV

**Figure 4:**
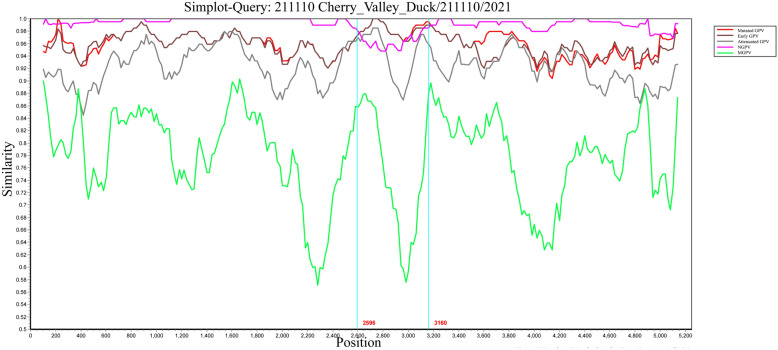
**Recombination analysis of the Cherry Valley Duck/China/211110 strain**. The genome was aligned and analyzed using MAFFT and Simplot v3.5.1. A potential recombination region is highlighted by the blue box. Major parental and related lineages (Early GPV, Attenuated GPV, MGPV, NGPV, and MDGPV) are color-coded and indicated in the legend. Genetic subgroups are delineated by boxes.

To evaluate the genetic recombination of GPV prevalent strains in China, we analyzed the genomes of four representative isolates (Goose/China/GS221102/2022, Goose/China/1008/2019, Cherry Valley duck/X6/2018, and Cherry Valley duck/211110/2021) using multiple algorithms integrated in RDP4.0 (RDP, GENECONV, Bootscan, Chimaera, SiScan, MAXCHI, and 3Seq) and validated with Simplot 3.5.1. Analysis revealed significant recombination signals for isolate Cherry Valley duck/211110/2021 across all tests (*P* < 0.01). RDP4.0 multi-algorithm analysis indicated a potential recombination region within the 2530–3014 nt interval of its genome. Phylogenetic analysis identified its primary parental source as the NGPV representative strain SDHT16 and the secondary as strain 06–0329 from the Early GPV branch. Simplot validation supported this finding: isolate Cherry Valley duck/211110/2021 showed high homology (> 95%) to Early GPV in the 2595–3160 nt region, whereas the remaining genomic regions exhibited highest similarity to NGPV (Additional file 5 and Figure 4). In contrast, no statistically significant recombination signals were detected for isolates Goose/China/GS221102/2022, Goose/China/1008/2019, and Cherry Valley duck/X6/2018 using RDP4.0 (*P* > 0.05), and no consistent recombination breakpoints were observed in Simplot analyses, indicating these three viruses did not undergo genome recombination (Additional file 6).

### Analysis of GPV and waterfowl circovirus co-circulation

**Figure 5:**
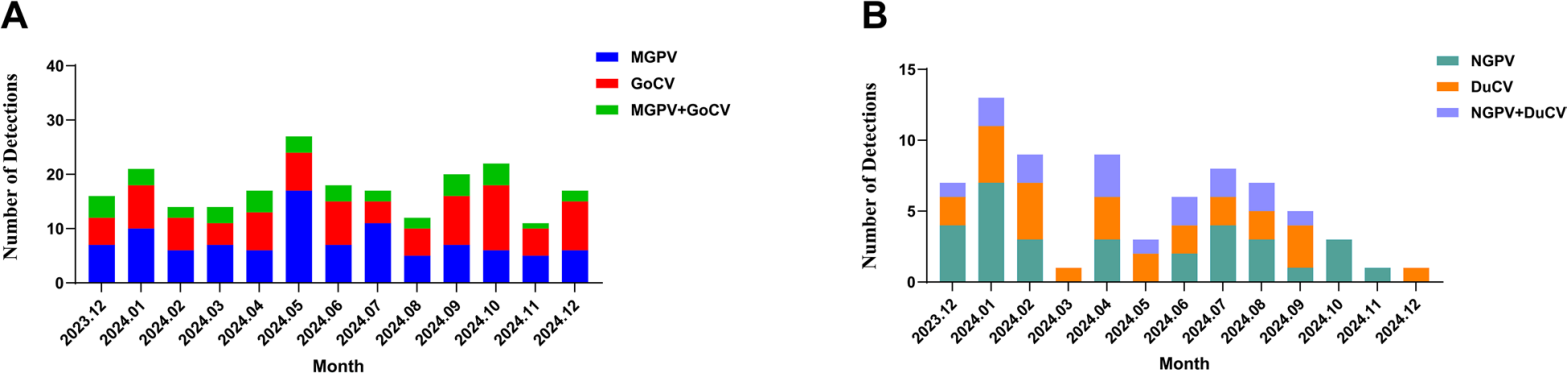
**Mixed infection analysis of waterfowl circovirus**.** A** The bar chart illustrates the monthly distribution of single and mixed infections involving Mutated GPV and GoCV. Infections are represented as follows: Mutated GPV (blue), GoCV (red), and Mutated GPV + GoCV (green). **B** The bar chart illustrates the monthly distribution of single and mixed infections involving NGPV and DuCV. Infections are represented as follows: NGPV (teal), DuCV (orange), and NGPV + DuCV (purple).

Mixed viral infections are common in waterfowl, with co-circulation of GPV and waterfowl circovirus being particularly prevalent and exacerbating pathogenicity in these birds. This study monitored GPV and waterfowl circovirus co-circulation in waterfowl from December 2023 to December 2024. A total of 370 samples were tested, comprising 286 from geese and 84 from ducks. In goose-derived samples, co-infection with GPV and GoCV was predominant. The GPV positivity rate was 35.0% (100/286), peaking at 63.0% (17/27) in May 2024. GoCV infection remained relatively stable with a positivity rate of 31.1% (89/286), peaking at 48.0% (12/25) in October 2024. The combined GPV and GoCV co-infection rate was 12.9% (37/286) (Figure 5A). Duck-derived samples primarily showed mixed infection with NGPV and DuCV. The NGPV positivity rate was 36.9% (31/84), while the DuCV positivity rate was 30.1% (26/84), resulting in a co-infection rate of 19.0% (16/84) (Figure 5B). These findings indicate a persistent co-circulation dynamic between GPV and waterfowl circovirus, specifically GPV/GoCV in geese and NGPV/DuCV in ducks.

### Genetic evolution analysis of waterfowl circovirus

**Figure 6:**
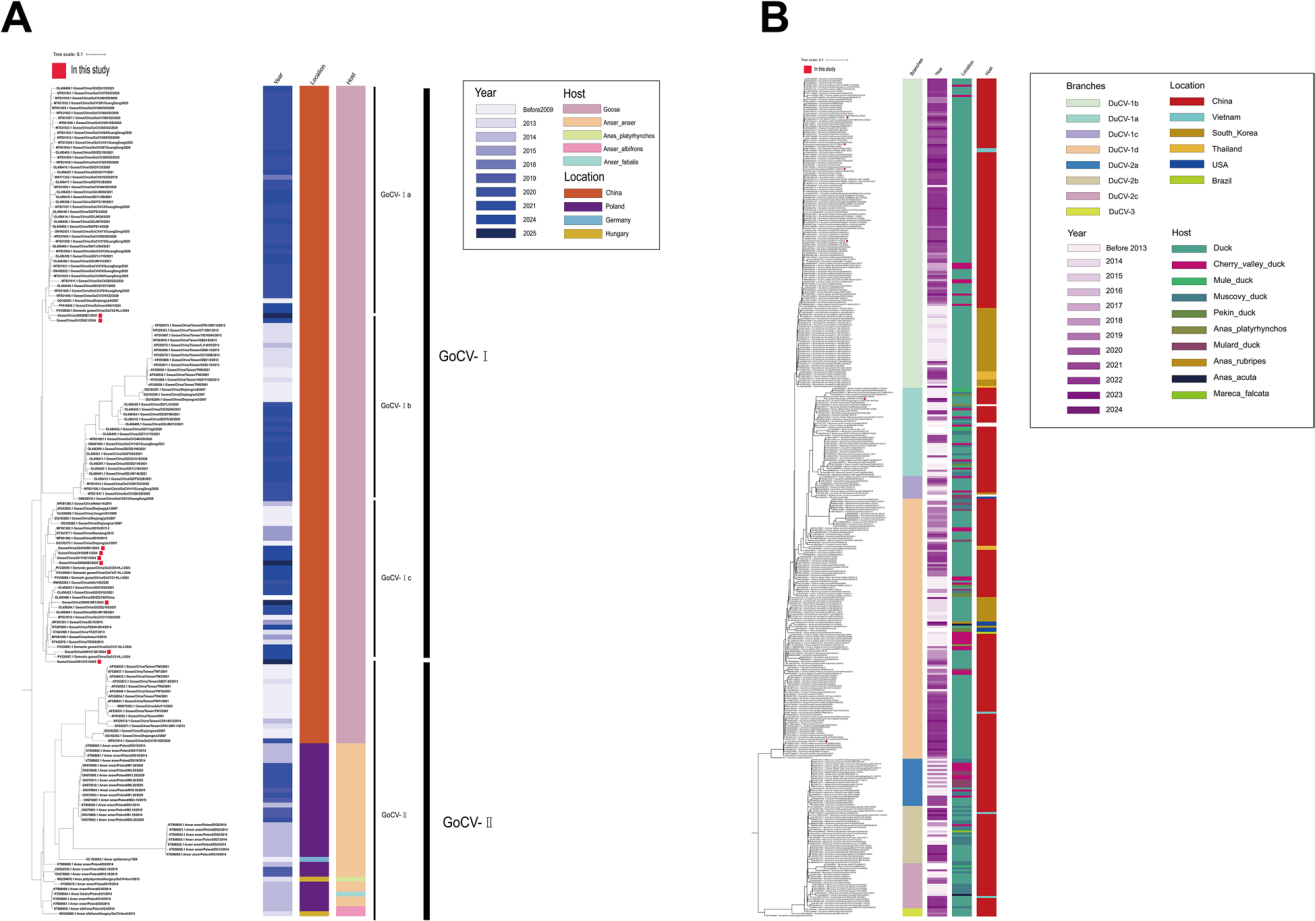
**Phylogenetic analysis of waterfowl circovirus**.** A** Phylogenetic analysis of GoCV whole genome. The nine GoCV isolates from this study are marked with red dots. The colored and black bars adjacent to the strain names represent their metadata: Year, Location, Host, and Genotype, respectively. **B** Phylogenetic analysis of DuCV whole genome. The six DuCV isolates from this study are marked with red dots. The colored bars adjacent to the strain names represent their metadata: Branches, Year, Location, and Host, respectively.

To investigate the prevalence of waterfowl circovirus in China, we performed whole-genome sequencing and evolutionary analysis on GoCV and DuCV isolates. The results of GoCV analysis revealed two distinct GoCV branches (GoCV-I and GoCV-II). GoCV-I further divided into three subtypes (GoCV-Ia, Ib, and Ic). All nine GoCV isolates from geese belonged to GoCV-I, with two classified as GoCV-Ia (Goose/China/250205E1, Goose/China/241208E1) and the remaining seven as GoCV-Ic (Goose/China/241030E1, Goose/China/241020E1, Goose/China/1110E1, Goose/China/0205E3, Goose/China/25H0216E1, Goose/China/24H1213E1, and 250121E1). Global prevalence analysis indicated that GoCV-I primarily circulates in Chinese geese: GoCV-Ia was mainly detected in Guangdong (2020–2025), GoCV-Ib was restricted to Taiwan (2013–2014), and GoCV-Ⅰc was widespread across multiple regions in China. In contrast, GoCV-II formed an independent transmission chain, initially found in Taiwanese geese (2001–2014) and later in Polish Anser anser (2014–2020), demonstrating notable cross-species adaptation potential (Figure 6A).

Phylogenetic analysis based on the whole genome of DuCV revealed eight genotypes, namely DuCV-1a, 1b, 1c, 1d, 2a, 2b, 2c, and 3. The six DuCV isolates obtained in our laboratory were all positioned within the DuCV-1 branch. Specifically, four isolates (Duck/China/Jiangsu/240801Y1, Duck/China/Jiangsu/231211Y1, Duck/China/Jiangsu/240601Y1, Duck/China/Jiangsu/0621Y1) belonged to genotype 1b; one isolate (Duck/China/Jiangsu/240403Y1/2024) belonged to genotype 1a; and one isolate (Duck/China/Jiangsu/240321Y1/2024) belonged to genotype 1d. Subsequently, genetic evolutionary analysis of published DuCV strains from NCBI revealed significant epidemic diversity. Specifically, DuCV-1a was predominantly prevalent in Fujian, Jiangsu, and Taiwan provinces of China, primarily infecting Muscovy duck, Mule duck, and Cherry valley duck. DuCV-1b circulated mainly in China, Vietnam, South Korea, and Thailand, with its hosts predominantly being Duck and Cherry valley duck. DuCV-1c was identified in China, South Korea, Thailand, and the United States, mainly infecting Mulard duck and Duck. DuCV-1d exhibited a broader distribution, being prevalent in China, South Korea, Thailand, and the United States, and infecting a wider range of hosts including Duck, Cherry valley duck, Muscovy duck, and wild waterfowl (e.g., Anas platyrhynchos). DuCV-2a/2b/2c were primarily prevalent in mainland China, with sustained circulation in recent years in Guangdong and Fujian provinces, and also detected to a certain extent in Vietnam and Taiwan, mainly infecting Muscovy duck. DuCV-3 was currently less frequently detected, with limited prevalence, mainly existing in mainland China, and primarily infecting Duck (Figure 6B).

### Analysis of nucleotide similarity of waterfowl circovirus

**Figure 7:**
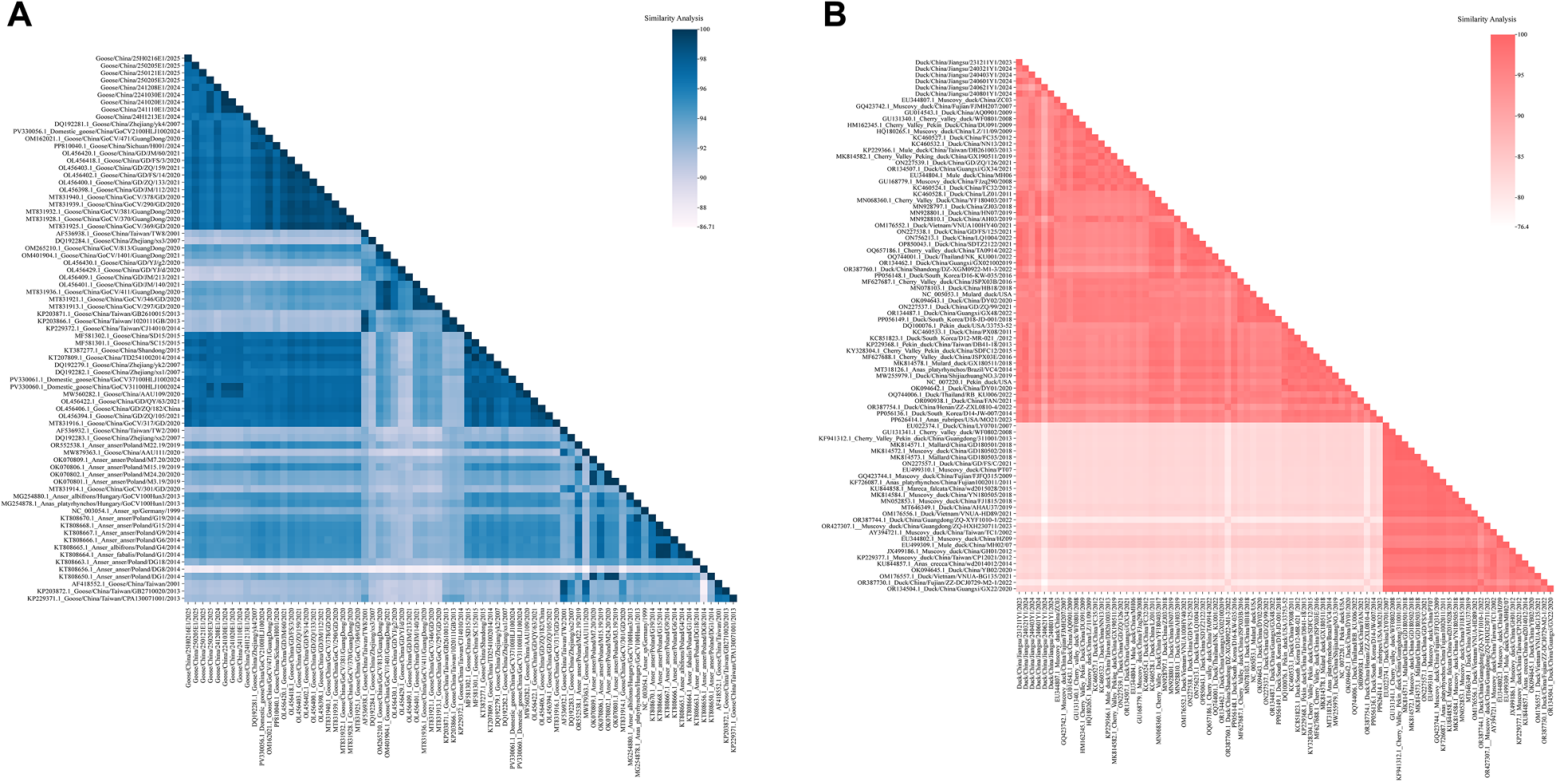
**Genomic nucleotide similarity analysis of waterfowl circovirus**.** A** Genomic nucleotide similarity analysis of GoCV. A heatmap illustrates the pairwise whole-genome similarity between the nine GoCV strains from this study and the reference strain. The color scale (top right) indicates similarity percentages from 86.71% to 100%. **B** Genomic nucleotide similarity analysis of DuCV. A heatmap illustrates the pairwise whole-genome similarity between the six DuCV strains from this study and the reference strain. The color scale (top right) indicates similarity percentages from 76.4% to 100%.

To analyze the genetic variation of waterfowl circovirus, we performed whole-genome nucleotide sequence identity comparisons between isolates from our laboratory and reference strains. The nucleotide alignment results for GoCV showed that the nucleotide identity of the isolates was highly consistent with the topological structure of the previous phylogenetic tree. The nucleotide identity of eight isolates (0216E1, 0205E1, 0121E1, 0205E3, 1030E1, 1020E1, 1110E1, 1213E1) to GoCV-Ic reference strains ranged from 95.88% to 99.07%. In contrast, the 1208E1 isolate exhibited a significantly different evolutionary placement, with a similarity of only 97.91% to 98.08% to GoCV-Ia reference strains. The nucleotide similarity among all reference strains ranged from 86.71% to 99.95%. Notably, the similarity among Chinese goose-derived strains was generally > 95%, suggesting high conservation of recently circulating strains in China (Figure 7A). Furthermore, the nucleotide alignment results for DuCV revealed significant branch-specificity in the evolutionary placement of the isolates. The nucleotide identity of the 240403Y1 isolate to DuCV-1a reference strains was 95.17%–97.33%; the identity of four isolates (231211Y1, 240601Y1, 240621Y1, 240801Y1) to the DuCV-1b reference strain ranged from 93.63% to 98.97%, with the lowest value (93.63%) observed for the 231211Y1 isolate, suggesting a possible recombination event; the identity of the 240,321 strain to DuCV-1d reference strains was 94.92%–98.77%. The nucleotide similarity among all DuCV reference strains ranged from 76.4% to 99.9 (Figure 7B). Therefore, these results indicate that waterfowl circovirus continue to undergo variation and recombination.

## Discussion

GPV, an important infectious disease affecting waterfowl farming industry, poses a serious threat to waterfowl and often leads to delayed growth and development [6, 35]. Therefore, the prevalence and genetic evolution of GPV have long been a major concern, but current systematic epidemiological investigations still fall short. GPV exhibits high genetic diversity and has evolved into two main branches: the classical GPV branch and the NGPV branch. This study revealed the complex characteristics of GPV prevalence in China and globally through continuous monitoring from 2018 to 2024, combined with phylogenetic analysis of both the VP1 gene and the whole genome. In recent years, GPVs isolated in China have shown clear host preferences: most strains from geese are MGPV, while most from ducks are NGPV. This discovery aligns with the previously reported phenomenon of GPV genotype replacement, where MGPV has become the dominant genotype in goose flocks, while NGPV has done so in duck flocks [36]. The fact that MGPV constitutes the highest proportion among goose isolates in the NCBI database also confirms this trend, indicating that MGPV has become the dominant genotype in the current goose population globally. The replacement of this genotype may be due to the selection pressure or adaptive evolution under vaccine immune pressure [16, 37, 38]. This could give MGPV an advantage in pathogenicity or transmission ability.

The expansion of the host range represents another major aspect in the evolution of GPV epidemics. Our study revealed that NGPV is gradually becoming the dominant circulating genotype in ducks, indicating the successful adaptation of GPV to duck. Furthermore, the fact that GPV has been isolated from wild birds—including swans and ostriches—indicates its ability to overcome species barriers [20–22]. Notably, gene mutations and recombination were observed in these isolates, suggesting that genetic variation in GPV is a prerequisite for cross-species transmission and warrants attention. China’s poultry industry is characterized by the coexistence of rural household rearing, commercial farms, and modern intensive ranches. These high-density, mixed-species systems create unique ecological niches for viral transmission [39]. This unique poultry farming practices, combined with the presence of migratory birds, facilitate virus transmission and host adaptation [40, 41]. This study indicated that MGPV is primarily prevalent in densely populated goose farming areas like Jiangsu, whereas NGPV holds a dominant position in duck populations within Shandong and Guangdong. This geographical distribution mirrors China’s regional breeding patterns, as these areas are characterized by abundant water resources and a high density of waterfowl farming. The prevalence of such intensive farming creates ideal conditions for viral transmission, driving NGPV to evolve host adaptation mechanisms that optimize replication in these environments. Consequently, this adaptation facilitates the virus’s persistence and circulation within major industrial clusters [40, 42]. Internationally, GPV infections have been documented in Southeast Asia and Europe, indicating a broader geographical distribution. The potential for cross-species transmission identified in this study could expand GPV’s host range, potentially accelerating its global dissemination. Regarding historical spread, phylogenetic analyses suggest that GPV may have disseminated from China to European countries (e.g., Hungary and Poland) and Southeast Asia. Consistent with previous reports [16], Hungary may have served as an epidemiological hub for regional transmission, though the precise routes of introduction remain to be fully elucidated. The complex long-distance migration of migratory birds deserves serious attention, as they may act as intermediate hosts and play a significant role in the cross-regional transmission of viruses. This is evidenced by the spread of avian influenza viruses and coronaviruses [43–45]. In line with these observations, our findings aligned with global trends indicating GPV genotype replacement, host range expansion, and geographic spread. In addition, this study verified through whole genome analysis that its results were consistent with the analysis of the VP1 gene, further demonstrating the effectiveness of the VP1 gene as a GPV typing marker. The MGPV and NGPV epidemic strains isolated in our laboratory exhibited over 95% genome similarity with the reference strain. This suggests that GPV genomes prevalent in China in recent years are relatively conserved. This finding further corroborates the results of phylogenetic analysis, indicating a high genetic correlation between these isolates and their respective branches. In contrast, the global distribution of GPV genome nucleotide similarity was considerably wider. The significant differences were observed between strains from different host sources, which may be linked to host-adaptive evolution, potentially reflecting distinct selective pressures exerted by various hosts on the virus. Recombination serves as a critical driving force behind virus evolution [46]. This study employed various algorithms, including RDP4.0, to perform recombination analysis on a subset of isolated strains. Notably, one NGPV isolate (the Cherry Valley duck/211110/2021 strain) exhibited significant recombination signals across multiple detection platforms. This isolate is likely the product of recombination between the NGPV subtype representative strain SDHT16 and the Early GPV representative strain 06–0329. This finding not only confirms the occurrence of recombination in GPVs but also suggests that recombination may play a crucial role in their genetic diversity and evolution, potentially giving rise to recombinant viruses with novel biological characteristics.

In addition to GPV, this study detected waterfowl circovirus (including GoCV and DuCV) and notably observed mixed infections of GPV with these two waterfowl circovirus types within waterfowl populations. As another important waterfowl pathogen, waterfowl circovirus poses a significant threat to waterfowl health [47]. The mixed infection state undoubtedly increases the complexity of waterfowl diseases, as interactions between different viruses—such as synergistic pathogenicity and immune suppression—may lead to more serious adverse consequences for the host than expected from a single viral infection. It has been confirmed that GPV synergistically enhances its viral replication and pathogenicity through co-infection with DuCV [48]. Furthermore, mixed infections of immunosuppressive viruses in poultry can damage the host’s immune defenses, thereby enhancing the overall immunosuppressive effect [49]. Although this study focused on genetic diversity, the detection of mixed infections indicates that when evaluating diseases like gosling plague, the potential influence of other circoviruses should not be overlooked. Genetic analyses of GoCV and DuCV revealed substantial diversity in waterfowl circovirus. GoCV strains diverged into distinct evolutionary branches (GoCV-Ⅰa and GoCV-Ⅰc), with some showing unique evolutionary paths, suggesting ongoing evolution. Similarly, DuCV isolates displayed branch-specific patterns, with low nucleotide consistency in the DuCV-1b branch (e.g., strain 231211Y1), which is suggestive of possible recombination events. The phylogenetic patterns observed in both GoCV and DuCV imply that recombination may play a broad role in shaping waterfowl virus diversity. In contrast, while GPV and waterfowl circovirus exhibited significant genetic diversity overall, goose-derived GPV strains in China shared high nucleotide similarity (> 95%), indicating a recent common ancestor and a relatively stable viral population. However, this apparent stability may conceal risks from recombination or the gradual accumulation of minor mutations.

In conclusion, this study shows that waterfowl viral diseases, especially GPV, are undergoing rapid evolution and changes in their epidemic patterns. The spread of different GPV genotypes, wider host range, regional differences, and viral recombination together pose global challenges. Mixed infections of GPV and waterfowl circovirus add complexity to disease diagnosis and control. These findings highlight the need for ongoing monitoring of viral genetic changes and disease spread, providing key evidence for creating more precise and forward-looking strategies to prevent and control waterfowl diseases.

## Supplementary Information

**Additional file** **1. Primer sequences for genome amplification of GPV and waterfowl circovirus.**

**Additional file** **2. PCR reaction system for GPV and waterfowl circovirus.**

**Additional file** **3. Primer-specific conditions for full-genome PCR amplification.**

**Additional file** **4. Details of the GPV and waterfowl circovirus isolates.**

**Additional file** **5. Whole-genome recombination analysis of GPV.** A whole-genome recombination analysis was performed using RDP4.0. **(A)** A major recombination breakpoint in the Cherry Valley Duck/China/211110 strain (indicated by the red box) was identified, with SDHT16 and 06-0329 as its parental strains.** (B)** A potential recombination event was detected in the Cherry Valley Duck/China/211110 strain (red) by phylogenetic analysis. Phylogenetic analysis identified 06-0329 (blue) and SDHT16 (green) as the primary and secondary parental strains, respectively.

**Additional file** **6. Genetic features of non-recombinant GPV strains. (A)** No recombination event was detected in the Cherry Valley duck/China/X6/2017 strain. It exhibits the closest genetic relationship to strains within the NGPV clade. **(B)** No recombination event was detected in the Goose/China/1008/2019 strain, which shows the highest similarity to the MGPV clade. **(C)** No recombination event was detected in the Goose/China/GS221102/2022 strain, indicating it is most closely related to the MGPV clade. In all Simplot analyses, reference strains are color-coded as follows: Early GPV (brown), Attenuated GPV (gray), MGPV (red), NGPV (purple), and MDPV (green).

## Data Availability

The obtained sequences in this study were submitted to GenBank under the accession number shown in Additional file 4.
