# Supplementary material for: Genomic surveillance and evolution of co-circulating goose parvovirus and waterfowl circovirus in China
Source: Vet Res. 2026 Jun 2;57:99. doi: 10.1186/s13567-026-01737-7 (PMC13231610; doi:10.1186/s13567-026-01737-7)
Supplement: Supplementary file 1 — Additional file 1. Primer sequences for genome amplification of GPV and waterfowl circovirus. [file 13567_2026_1737_MOESM1_ESM.docx]

**Additional file 1.** Primer sequences for genome amplification of GPV and waterfowl circovirus

| Primers | Sequence (5’-3’) |
| --- | --- |
| GPV-F1 | CTYATTGKAGGGTTCGTTCR |
| GPV-R1 | GCATGCGYGYGGTCAACMTAA |
| GPV-F2 | GCATGCGCGCGGTCAGCCCAATRGTTAA |
| GPV-R2 | AGTTGAAAGAAGAAGTCCGTTTCGTCCT |
| GPV-F3 | ATGGCACTTTCTAGGCCTCTKCA |
| GPV-R3 | ACGAGGCGGCTGCAGTYTCATA |
| GPV-F4 | TCCTACTAGGGAGGAGTTAGAAGA |
| GPV-R4 | TGRACATTGAAGATCTTRAATTTAAGRG |
| GPV-F5 | ATTCCCAATGGATGGGAAACACAGT |
| GPV-R5 | CAGATTTTGAGTTAGATATCTGRTTCCA |
| GPV-F6 | GTTCTTGGAGCTTTACCAGGAAT |
| GPV-R6 | GYTGGCCRCRCGCATGCG |
| GPV VP1-F | AARATGAACTTGCAGAAA |
| GPV VP1-R | AGGAAGTGYTTTATTTGA |
| GoCV F1 | ACCGGCGCYTGTACTCCGTA |
| GoCV R1 | AATAATATATACGGCGCYTGTAACG |
| GoCV F2 | TCACCGACTCGAAGGTATGTCGAC |
| GoCV R2 | TATTCGTCCGATGTGTAGCCTTCG |
| DuCV F1 | ACGCTCGACAATTGCAAGTTY |
| DuCV R1 | CCTMTCGGYGTSCATATCGT |
| DuCV F2 | CATGTAGCTRTTYGTGTCY |
| DuCV R2 | GAGAGCCAGGCTCTTCCT |

To amplify the target sequences, specific primer sets were employed. The GPV VP1 gene was amplified using the primer set GPV VP1-F/R. For complete genome sequencing, the following primer sets were used: GPV F1/R1, F2/R2, F3/R3, F4/R4, F5/R5, and F6/R6 for the GPV genome; GoCV F1/R1 and F2/R2 for the GoCV genome; and DuCV F1/R1 and F2/R2 for the DuCV genome.
