# Supplementary material for: Genomic surveillance and evolution of co-circulating goose parvovirus and waterfowl circovirus in China
Source: Vet Res. 2026 Jun 2;57:99. doi: 10.1186/s13567-026-01737-7 (PMC13231610; doi:10.1186/s13567-026-01737-7)
Supplement: Supplementary file 2 — Additional file 2. PCR reaction system for GPV and waterfowl circovirus. [file 13567_2026_1737_MOESM2_ESM.docx]

**Additional file 2.** PCR reaction system for GPV and waterfowl circovirus

| Reagent | Volume |
| --- | --- |
| 2×Taq Plus Master Mix Ⅱ | 10μL |
| Forward primer | 1μL |
| Reverse primer | 1μL |
| DNA | 2μL |
| ddH_2_O | 6μL |
| Total | 20μL |
