# Supplementary material for: Genomic surveillance and evolution of co-circulating goose parvovirus and waterfowl circovirus in China
Source: Vet Res. 2026 Jun 2;57:99. doi: 10.1186/s13567-026-01737-7 (PMC13231610; doi:10.1186/s13567-026-01737-7)
Supplement: Supplementary file 3 — Additional file 3. Primer-specific conditions for full-genome PCR amplification. [file 13567_2026_1737_MOESM3_ESM.docx]

**Additional file 3.** Primer-specific conditions for full-genome PCR amplification.

| Virus | Primer pair | Annealing temperature (℃) | Extension time (sec) |
| --- | --- | --- | --- |
| GPV | F1/R1 | 50 | 5 |
|  | F2/R2 | 53 | 5 |
|  | F3/R3 | 52 | 15 |
|  | F4/R4 | 50 | 10 |
|  | F5/R5 | 51 | 10 |
|  | F6/R6 | 54 | 5 |
| GoCV | GoCV-F1/R1 | 58 | 10 |
|  | GoCV-F2/R2 | 55 | 10 |
| DuCV | DuCV-F1/R1 | 55 | 10 |
|  | DuCV-F2/R2 | 52 | 10 |
