# Supplementary material for: Genomic surveillance and evolution of co-circulating goose parvovirus and waterfowl circovirus in China
Source: Vet Res. 2026 Jun 2;57:99. doi: 10.1186/s13567-026-01737-7 (PMC13231610; doi:10.1186/s13567-026-01737-7)
Supplement: Supplementary file 4 — Additional file 4. Details of the GPV and waterfowl circovirus isolates. [file 13567_2026_1737_MOESM4_ESM.docx]

**Additional file 4.** Details of the GPV and waterfowl circovirus isolates

| Name | Host | Year | Location | Branches | Accession number |
| --- | --- | --- | --- | --- | --- |
| Goose/Jiangsu/C5/2020 | Goose | 2020 | Jiangsu | Mutated GPV | PX374748 |
| Goose/China/23h0714E1/2023 | Goose | 2023 | Jiangsu | Mutated GPV | PX374759 |
| Goose/China/YZ8/2018 | Goose | 2018 | Jiangsu | Mutated GPV | PX374739 |
| Goose/China/YZ7/2018 | Goose | 2018 | Jiangsu | Mutated GPV | PX374741 |
| Goose/Shandong/LC/2020 | Goose | 2020 | Shandong | Mutated GPV | PX374738 |
| Goose/China/AH6/2018 | Goose | 2018 | Anhui | Mutated GPV | PX374774 |
| Goose/China/230531E1/2023 | Goose | 2023 | Jiangsu | Mutated GPV | PX374761 |
| Goose/China/AH12/2018 | Goose | 2018 | Anhui | Mutated GPV | PX374745 |
| Goose/Jiangsu/C2/2019 | Goose | 2019 | Jiangsu | Mutated GPV | PX374742 |
| Goose/China/220720/2022 | Goose | 2022 | Jiangsu | Mutated GPV | PX374767 |
| Goose/China/0510/2019 | Goose | 2019 | Jiangsu | Mutated GPV | PX374744 |
| Goose/Shandong/D2/2019 | Goose | 2019 | Shandong | Mutated GPV | PX374746 |
| Goose/China/210509/2021 | Goose | 2021 | Jiangsu | Mutated GPV | PX374747 |
| Goose/China/240704E2/2024 | Goose | 2024 | Jiangsu | Mutated GPV | PX374783 |
| Goose/China/241124E1/2024 | Goose | 2024 | Jiangsu | Mutated GPV | PX374795 |
| Goose/Jiangsu/C1/2019 | Goose | 2019 | Jiangsu | Mutated GPV | PX374743 |
| Goose/China/24H1207E1/2024 | Goose | 2024 | Jiangsu | Mutated GPV | PX374787 |
| Goose/China/24H0908E1/2024 | Goose | 2024 | Jiangsu | Mutated GPV | PX374786 |
| Goose/China/241109E1/2024 | Goose | 2024 | Jiangsu | Mutated GPV | PX374794 |
| Goose/China/240613E1/2024 | Goose | 2024 | Jiangsu | Mutated GPV | PX374782 |
| Goose/Shandong/XP/2020 | Goose | 2020 | Shandong | Mutated GPV | PX374752 |
| Goose/Jiangsu/C3/2019 | Goose | 2019 | Jiangsu | Mutated GPV | PX374751 |
| Goose/Jiangsu/C9/2019 | Goose | 2019 | Jiangsu | Mutated GPV | PX374749 |
| Goose/Jiangsu/C10/2019 | Goose | 2019 | Jiangsu | Mutated GPV | PX374750 |
| Goose/China/YZ3/2018 | Goose | 2018 | Jiangsu | Mutated GPV | PX374758 |
| Goose/China/210132/2021 | Goose | 2021 | Jiangsu | Mutated GPV | PX374756 |
| Goose/China/210156/2021 | Goose | 2021 | Jiangsu | Mutated GPV | PX374757 |
| Goose/Jiangsu/C6/2020 | Goose | 2020 | Jiangsu | Mutated GPV | PX374753 |
| Goose/China/220201/2021 | Goose | 2021 | Jiangsu | Mutated GPV | PX374762 |
| Goose/China/2401001E1/2024 | Goose | 2024 | Jiangsu | Mutated GPV | PX374788 |
| Goose/China/241005E2/2024 | Goose | 2024 | Jiangsu | Mutated GPV | PX374792 |
| Goose/China/23H0422E1 | Goose | 2023 | Jiangsu | Mutated GPV | PX374764 |
| Goose/China/240930E1/2024 | Goose | 2024 | Jiangsu | Mutated GPV | PX374791 |
| Goose/China/23H0611E2 | Goose | 2023 | Jiangsu | Mutated GPV | PX374763 |
| Goose/China/YZ4/2018 | Goose | 2018 | Jiangsu | Mutated GPV | PX374754 |
| Goose/China/YZ5/2018 | Goose | 2018 | Jiangsu | Mutated GPV | PX374755 |
| Goose/China/241007E3/2024 | Goose | 2024 | Jiangsu | Mutated GPV | PX374784 |
| Goose/China/240924E1/2024 | Goose | 2024 | Jiangsu | Mutated GPV | PX374790 |
| Goose/China/230446/2023 | Goose | 2023 | Jiangsu | Mutated GPV | PX374765 |
| Goose/China/24H0525E1/2024 | Goose | 2024 | Jiangsu | Mutated GPV | PX374785 |
| Goose/China/241023E1/2024 | Goose | 2024 | Jiangsu | Mutated GPV | PX374793 |
| Goose/Jiangsu/C8/2019 | Goose | 2019 | Jiangsu | Mutated GPV | PX374736 |
| Goose/China/23H0712E1/2023 | Goose | 2023 | Jiangsu | Mutated GPV | PX374766 |
| Goose/China/YZ2/2017 | Goose | 2017 | Jiangsu | Mutated GPV | PX374740 |
| Goose/China/220728/2022 | Goose | 2022 | Jiangsu | Mutated GPV | PX374760 |
| Goose/China/220730/2022 | Goose | 2022 | Jiangsu | Mutated GPV | PX374768 |
| Goose/China/220733/2022 | Goose | 2022 | Jiangsu | Mutated GPV | PX374769 |
| Goose/China/2401008E1/2024 | Goose | 2024 | Jiangsu | Mutated GPV | PX374789 |
| Goose/China/230618E2/2023 | Goose | 2023 | Jiangsu | Mutated GPV | PX374771 |
| Goose/China/GS221102/2022 | Goose | 2022 | Shandong | Mutated GPV | PX374776 |
| Goose/China/23H0511E1/2023 | Goose | 2023 | Jiangsu | Mutated GPV | PX374770 |
| Goose/China/GS221101/2022 | Goose | 2022 | Shandong | Mutated GPV | PX374775 |
| Goose/China/AH7/2018 | Goose | 2018 | Anhui | Early GPV | PX374772 |
| Goose/China/AH10/2018 | Goose | 2018 | Anhui | Early GPV | PX374773 |
| Goose/China/220408/2022 | Goose | 2022 | Jiangsu | Attenuated GPV | PX374779 |
| Goose/Jiangsu/JS/2020 | Goose | 2020 | Jiangsu | Attenuated GPV | PX374781 |
| Goose/Jiangsu/YC/2020 | Goose | 2020 | Jiangsu | Attenuated GPV | PX374737 |
| Goose/China/YZ1/2017 | Goose | 2017 | Jiangsu | Attenuated GPV | PX374780 |
| Goose/China/220214/2022 | Goose | 2022 | Jiangsu | Attenuated GPV | PX374777 |
| Goose/China/220407/2022 | Goose | 2022 | Jiangsu | Attenuated GPV | PX374778 |
| Duck/China/211110/2021 | Duck | 2021 | Jiangsu | NGPV | PX374800 |
| Duck/China/210123/2021 | Duck | 2021 | Jiangsu | NGPV | PX374796 |
| Duck/China/220712/2022 | Duck | 2022 | Anhui | NGPV | PX374804 |
| Duck/China/211116/2021 | Duck | 2021 | Jiangsu | NGPV | PX374801 |
| Duck/China/220709/2022 | Duck | 2022 | Jiangsu | NGPV | PX374803 |
| Duck/China/230418/2023 | Duck | 2023 | Jiangsu | NGPV | PX374805 |
| Muscovy_duck/China/JX24/2023 | Muscovy Duck | 2023 | Jiangxi | NGPV | PX374811 |
| Muscovy_duck/China/JX26/2023 | Muscovy Duck | 2023 | Jiangxi | NGPV | PX374810 |
| Muscovy_duck/China/JX21/2023 | Muscovy Duck | 2023 | Jiangxi | NGPV | PX374808 |
| Muscovy_duck/China/JX23/2023 | Muscovy Duck | 2023 | Jiangxi | NGPV | PX374809 |
| Cherry_Valley_duck/Shandong/X6/2017 | Cherry Valley duck | 2017 | Shandong | NGPV | PX374798 |
| Duck/China/X5/2017 | Duck | 2017 | Shandong | NGPV | PX374797 |
| Cherry_Valley_duck/China/211109/2021 | Cherry Valley duck | 2021 | Jiangsu | NGPV | PX374802 |
| Cherry_Valley_duck/China/X7/2017 | Cherry Valley duck | 2017 | Shandong | NGPV | PX374799 |
| Muscovy_duck/China/JX28/2023 | Muscovy duck | 2023 | Jiangxi | NGPV | PX374812 |
| Duck/Shandong/X1/2018 | Duck | 2018 | Guangdong | MDPV | PX374806 |
| Duck/Guangdong/X2/2017 | Duck | 2017 | Guangdong | MDPV | PX374807 |
| Goose/China/250205E1 | Goose | 2025 | Jiangsu | GoCV-1a | PX374733 |
| Goose/China/241208E1 | Goose | 2024 | Jiangsu | GoCV-1a | PX374730 |
| Goose/China/241030E1 | Goose | 2024 | Jiangsu | GoCV-1c | PX374728 |
| Goose/China/241020E1 | Goose | 2024 | Jiangsu | GoCV-1c | PX374727 |
| Goose/China/241110E1 | Goose | 2024 | Jiangsu | GoCV-1c | PX374729 |
| Goose/China/250205E3 | Goose | 2025 | Jiangsu | GoCV-1c | PX374734 |
| Goose/China/25H0216E1 | Goose | 2025 | Jiangsu | GoCV-1c | PX374735 |
| Goose/China/24H1213E1 | Goose | 2024 | Jiangsu | GoCV-1c | PX374731 |
| Goose/China/250121E1 | Goose | 2025 | Jiangsu | GoCV-1c | PX374732 |
| Duck/China/Jiangsu/240403Y1 | Duck | 2024 | Jiangsu | DuCV-1a | PX459238 |
| Duck/China/Jiangsu/240801Y1 | Duck | 2024 | Jiangsu | DuCV-1b | PX725408 |
| Duck/China/Jiangsu/231211Y1 | Duck | 2023 | Jiangsu | DuCV-1b | PX374813 |
| Duck/China/Jiangsu/240601Y1 | Duck | 2024 | Jiangsu | DuCV-1b | PX374815 |
| Duck/China/Jiangsu/240621Y1 | Duck | 2024 | Jiangsu | DuCV-1b | PX374816 |
| Duck/China/Jiangsu/240321Y1 | Duck | 2024 | Jiangsu | DuCV-1d | PX374814 |
