# Supplementary figures and images for: Genomic surveillance and evolution of co-circulating goose parvovirus and waterfowl circovirus in China
Source: Vet Res. 2026 Jun 2;57:99. doi: 10.1186/s13567-026-01737-7 (PMC13231610; doi:10.1186/s13567-026-01737-7)

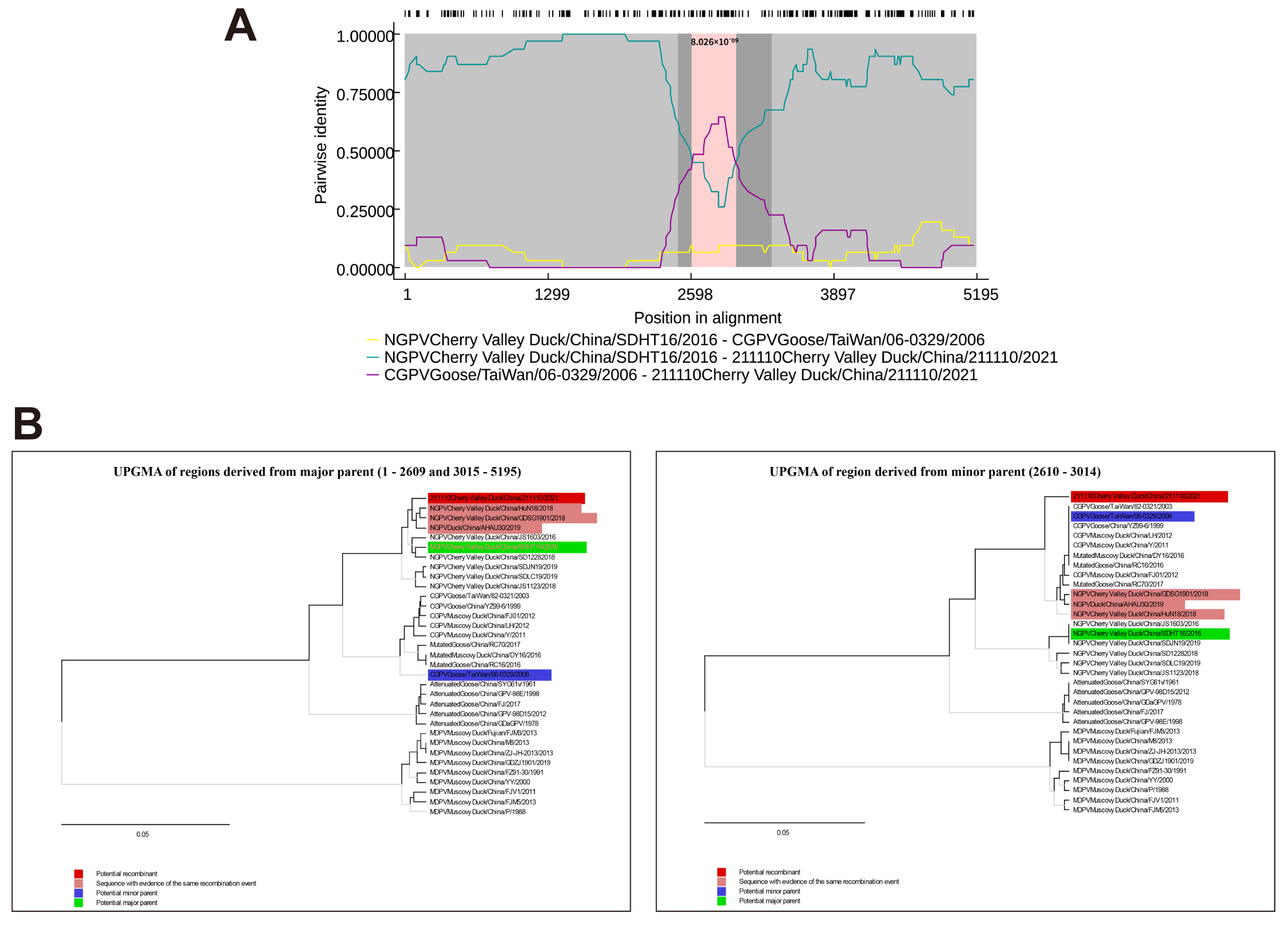

Supplement: Supplementary file 5 — Additional file 5. Whole-genome recombination analysis of GPV. A whole-genome recombination analysis was performed using RDP4.0. (A) A major recombination breakpoint in the Cherry Valley Duck/China/211110 strain (indicated by the red box) was identified, with SDHT16 and 06-0329 as its parental strains. (B) A potential recombination event was detected in the Cherry Valley Duck/China/211110 strain (red) by phylogenetic analysis. Phylogenetic analysis identified 06-0329 (blue) and SDHT16 (green) as the primary and secondary parental strains, respectively. [file 13567_2026_1737_MOESM5_ESM.tif]

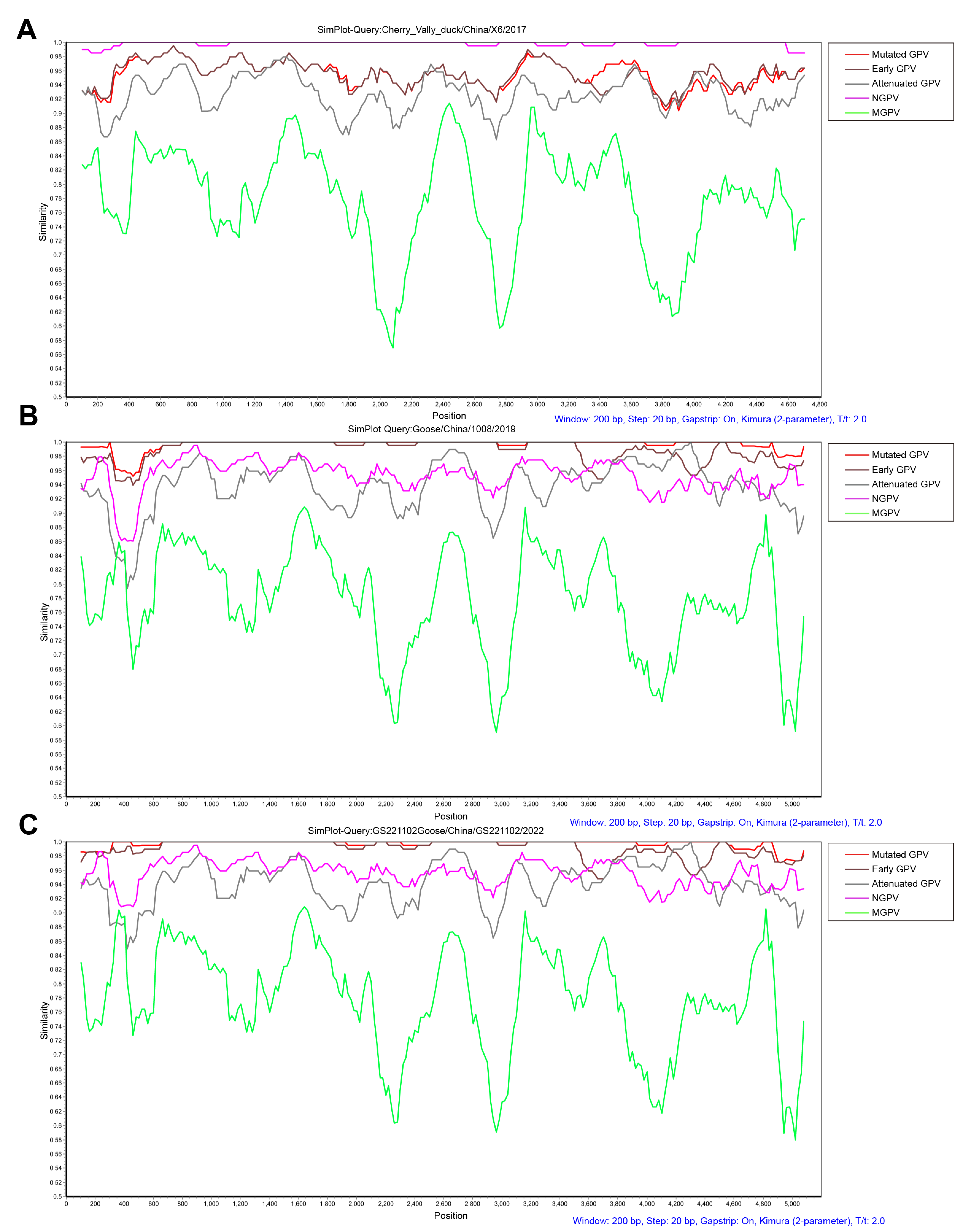

Supplement: Supplementary file 6 — Additional file 6. Genetic features of non-recombinant GPV strains. (A) No recombination event was detected in the Cherry Valley duck/China/X6/2017 strain. It exhibits the closest genetic relationship to strains within the NGPV clade. (B) No recombination event was detected in the Goose/China/1008/2019 strain, which shows the highest similarity to the MGPV clade. (C) No recombination event was detected in the Goose/China/GS221102/2022 strain, indicating it is most closely related to the MGPV clade. In all Simplot analyses, reference strains are color-coded as follows: Early GPV (brown), Attenuated GPV (gray), MGPV (red), NGPV (purple), and MDPV (green). [file 13567_2026_1737_MOESM6_ESM.tif]
